# Levothyroxine Therapy: Changes of TSH Levels by Switching Patients from Tablet to Liquid Formulation. A Systematic Review and Meta-Analysis

**DOI:** 10.3389/fendo.2018.00010

**Published:** 2018-01-26

**Authors:** Camilla Virili, Luca Giovanella, Poupak Fallahi, Alessandro Antonelli, Maria Giulia Santaguida, Marco Centanni, Pierpaolo Trimboli

**Affiliations:** ^1^Department of Medico-Surgical Sciences and Biotechnologies, Sapienza University of Rome, Latina, Italy; ^2^Department of Nuclear Medicine and Thyroid Centre, Oncology Institute of Southern Switzerland, Bellinzona, Switzerland; ^3^Department of Clinical and Experimental Medicine, University of Pisa, Pisa, Italy

**Keywords:** thyroxine absorption, oral liquid levothyroxine, thyroxine malabsorption, drugs dissolution, gastrointestinal disorders

## Abstract

**Background:**

In the last years, levothyroxine (LT4) has been commercialized also in liquid formulation, which is less sensitive to the factors known to reduce the absorption of tablet LT4. To date, there is no robust information that liquid LT4 can improve pharmacologic thyroid homeostasis of patients with reduced efficacy of tablet LT4. This analysis aimed at achieving solid evidence that switching thyroxine treatment from tablet to liquid preparation improves patients’ TSH levels.

**Methods:**

The search was performed in PubMed/MEDLINE and Scopus database based on the terms “thyroid,” “levothyroxine,” and “liquid,” and updated until September 25, 2017. Studies were included only if they described patients with suboptimal TSH on tablet LT4, subsequently switched to liquid LT4.

**Results:**

The literature search retrieved 462 articles and six were finally included. The pooled mean difference of TSH value between tablet and liquid LT4 was 4.23 mIU/L (95% CI from 3.69 to 4.77). Mild heterogeneity was found (I_2_ 60%). Overall mean difference of TSH was significant (*p* < 0.0001).

**Conclusion:**

The present meta-analysis showed that patients with suboptimal TSH on tablet LT4 can have a significantly improved TSH by switching to liquid LT4 formulation at unchanged dose.

## Introduction

### Rationale

Levothyroxine (LT4) is one of the most prescribed drugs worldwide and is characterized by a narrow therapeutic index (1). Therefore, this drug would require a careful dose individualization (2, 3) to avoid side effects of iatrogenic hyper- and hypothyroidism, even in their subclinical form (4). Generally, the efficacy of LT4 is evaluated by serum TSH value as it is considered the most accurate measurement of thyroid pharmacological homeostasis, and usually physician assess in each patient a specific TSH target to be reached and maintained. However, the desired TSH may be not reached in about one patient out of two (5). Indeed, even if a dose of 1.6–1.8 mcg/kg/day of LT4 should be satisfactory to treat hypothyroid patients (6), a non-negligible rate of them would require a dose increased up to 30–40% (7, 8). Several factors may influence the achievement of the target TSH. Some of these are patient/physician dependent, such as the prescribed dose, the LT4 intake schedule, and the patient’s compliance (9). In addition, several studies investigated systematically the LT4 ineffectiveness and demonstrated that intestinal disorders (i.e., celiac disease, lactose intolerance, dysbiosis) (10–13), gastric diseases (i.e., chronic atrophic gastritis, *Helicobacter pylori* infection) (7, 14), and concomitant intake of some drugs or ingestion of food or beverages (15, 16) can interfere with the LT4 absorption, thus reducing its efficacy. Notably, the failure to achieve the therapeutic target can determine multiple dosage adjustments, with consequent increase of health-care resources costs by up to 84% (17).

### Objectives

Almost all studies focusing on LT4 efficacy evaluated the performance of its tablet formulation, which is the traditional one. In the last decade, due to the abovementioned interferences, the pharmaceutical research developed new LT4 formulations, such as softgel capsule and liquid preparations, which have become available in a growing number of countries. These formulations are bioequivalent as compared to the traditional one but are characterized by different excipients; in fact, the active ingredient is dissolved in glycerin and surrounded by a gelatin shell (softgel capsule) or dissolved in glycerin and ethanol (liquid solution) (18). The main goal of softgel capsule and liquid solution use is to overcome the tablet LT4 dissolution problems (18, 19). This raised interesting outlook and some authors investigated the effectiveness of softgel and liquid LT4 with different study designs. Interestingly, liquid LT4 has been proven to be quickly absorbed because of its physical status: in fact, it does not require the dissolution phase, which is instead a necessary step for tablet (solid phase) absorption (19). Also, the issue whether the liquid LT4 efficacy is impaired by breakfast/coffee was investigated in six prospective studies (20–25), including one double-blind placebo controlled trial (24), one with a cross-over design (22), and one which evaluated the efficacy of the switch between liquid and softgel formulations (25); there, it was clearly demonstrated that liquid LT4 efficacy is not influenced by the lag time between breakfast and drug ingestion. On the contrary, the actual reliability of liquid LT4 in treating patients with not optimal TSH during therapy with traditional tablet formulation due to gastrointestinal diseases, polypharmacy, or not definitely demonstrated LT4 malabsorption remains not yet assessed. In fact, most of the studies showed heterogeneous designs in limited patients’ series, thus hampering the achievement of solid evidence.

### Research Question

Therefore, the lack of definite proofs that liquid LT4 can improve pharmacological thyroid homeostasis, prompted us to review the targeted literature. The present study was aimed at systematically review the literature to strengthen evidence that, in patients with reduced efficacy of tablet LT4, serum TSH can be normalized by switching therapy to liquid preparation. Based on the results found, we also attempted to perform a meta-analysis of retrieved data.

## Materials and Methods

### Study Design

The study design included studies investigating the efficacy of liquid LT4 in the treatment of hypothyroidism in two online databases.

### Participants, Interventions, Comparators

Participants were patients treated with tablet LT4 and having not optimal TSH, and switched to liquid LT4 with unchanged LT4 dose. There was no comparator.

### Search Strategy

A comprehensive computer literature search of the PubMed/MEDLINE and Scopus databases was conducted to find published articles on this topic. The search algorithm was based on the combinations of the terms “thyroid” AND “levothyroxine” AND “liquid.” A beginning date limit was not used, and the search was updated until September 21, 2017. No language restriction was adopted. To identify additional studies and expand our search, references lists of the retrieved articles could be screened. Two investigators (CV and PT) independently searched articles applying this strategy.

### Data Sources, Studies Sections, and Data Extraction

Original articles reporting experience on the use of liquid LT4 in humans were initially eligible for the systematic review. We selected studies according to the following criteria: studies were included if they reported patients with TSH levels higher than the upper reference value on tablet LT4 and then addressed to switch to liquid LT4. As exclusion criteria, studies were not included if they described a series of patients smaller than 10 cases (i.e., case report and cases series), used different dose of liquid LT4 with respect to that of tablet LT4, described the use of a non-pharmaceutically produced liquid preparation, included only patients requiring a specific TSH value (i.e., patients followed-up for differentiated thyroid carcinomas in whom TSH target may depend on the risk category of each case). In addition, articles with unclear data, and series with overlapping data were excluded. The same two authors (CV and PT) independently screened titles and abstracts of the retrieved articles according to the above criteria, reviewed the full-texts, and selected articles for their final inclusion. For each included study, information was extracted concerning study data: authors, year of publication, journal, study aim, study design, main results, and country of patients’ origin. Age of patients enrolled in the studies was also collected, when available.

### Data Analysis

Statistical analysis was performed using the Review Manager (RevMan) version 5.3 (Computer program; Copenhagen: The Nordic Cochrane Centre, The Cochrane Collaboration, 2014). According to the Cochrane handbook, statistical analysis was conducted as generic inverse variance, mean difference was considered as the effect measure and 95% confidence interval (95% CI) was calculated for totals and each study. According to these criteria, the mean difference and SE of TSH value on tablet LT4 minus that on liquid LT4 (at least 2 months therapy) was obtained for each group or subgroup included for the meta-analysis. When TSH data were not available in a PDF paper, the authors were contacted directly. Thus, the pooled TSH mean difference (and 95% CI) was calculated and the fixed effect model was used. I-square index was used to quantify the heterogeneity among the studies, and the absence of significant heterogeneity was defined as an I-square value <50%. Statistical significance of the overall result was analyzed by test for overall effect. The assessment of risk of bias was made by two authors (CV and PT) according to the Cochrane handbook; they evaluated the quality of the included studies based on their design, presence of carryover effect, data analysis performed, and reporting of results. Statistical significance was set at *p* < 0.05.

## Results

### Study Selection and Characteristics

**Figure 1:**
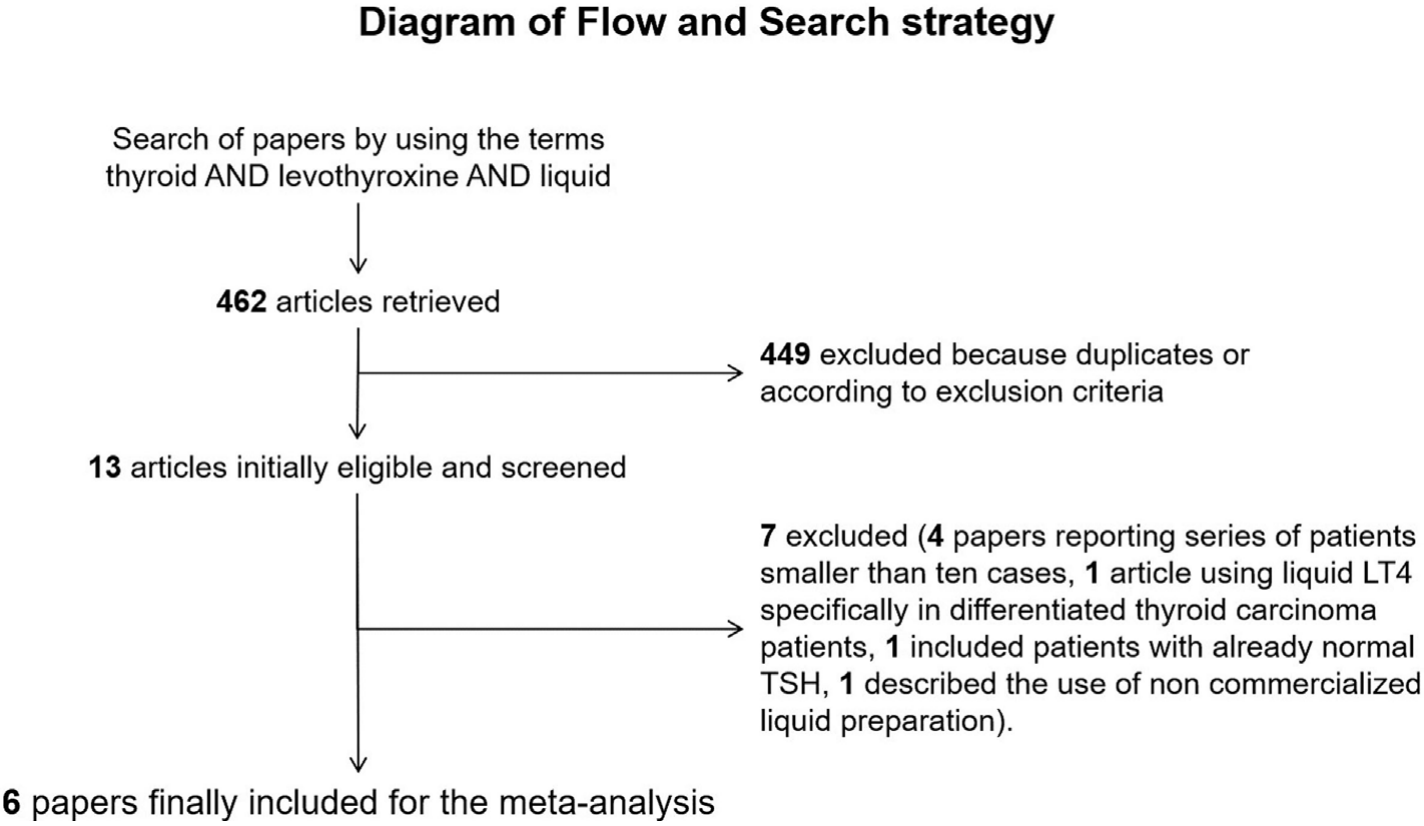
Diagram of flow of search strategy and results.

The comprehensive computer literature search retrieved 369 articles from PubMed/MEDLINE and 93 papers from Scopus database. Once excluded duplicates articles, the papers initially included were 13. Abstracts of these articles were screened and 7 (21, 26–31) were excluded according to the abovementioned criteria [4 reported small series (26, 28–30), 1 included patients showing the effect of switching T4 treatment in patients, which had already reached normal TSH with traditional tablet formulation (27), one described the use of a non-pharmaceutically produced liquid preparation (31), one enrolled only differentiated thyroid carcinoma patients (21)]. Finally, the systematic review included six original articles (32–37). Figure 1 illustrates the diagram of flow to retrieve the final series of papers.

### Synthesized Analysis

**Figure 2:**
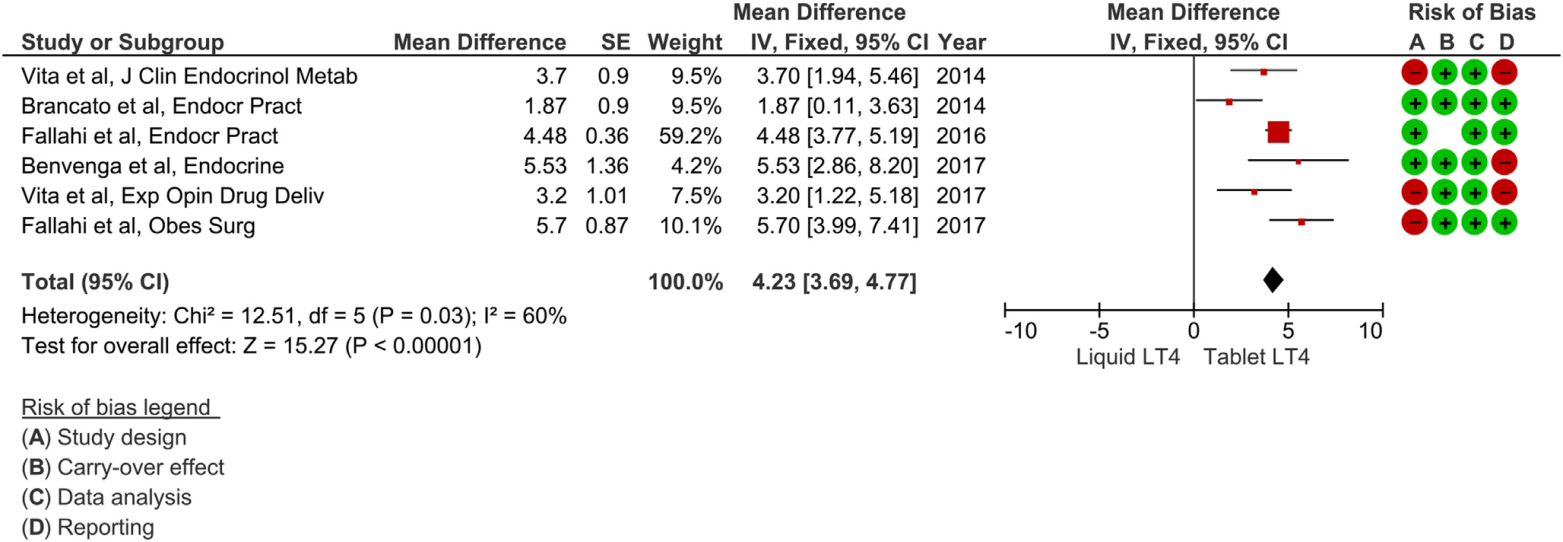
Forest plot of meta-analysis (fixed-effect) of six studies reporting patients who switched from tablet to liquid levothyroxine (LT4). Mean difference was referred to TSH levels (i.e., mean TSH on tablet LT4 minus mean TSH on liquid LT4). One study (36) described two subgroups of patients, and we excluded that of four cases according to the studies selection criteria (see Materials and Methods). Risk of bias assessment was represented as green when there was low risk, red when there was high risk, and white when the risk was unclear.

The included papers were published from 2014 to 2017 (32–37), and authors were Italian in all cases. All these studies had a prospective design; analyzed patients were already on tablet LT4 treatment without reaching optimal TSH values. Afterward, all subjects have been switched to liquid LT4 and the change of serum TSH values have been evaluated after 2 months. Overall, a number of 141 patients were described. Notably, all these studies modified LT4 therapy from tablet to liquid formulation with unchanged dose. Possible overlapping of data was excluded by contacting the authors. Table 1 summarizes the characteristics of these studies. The pooled mean difference of TSH value between tablet LT4 and liquid LT4 was 4.23 mIU/L (95% CI from 3.69 to 4.77). Considering the single studies, TSH levels were reduced after liquid LT4 in all cases with a mean difference from 1.87 to 5.70 mIU/L. Mild heterogeneity between the studies was found (I_2_ 60%). Overall mean difference of TSH was significant as showed by test of overall effect (*p* < 0.0001) (Figure 2).

**Table 1:** Overview of the studies included in the present review.

Reference	Year	Study type	Patients type	Patients who completed the study (*n*)
Vita et al. (32)	2014	Prospective	Patients taking levothyroxine (LT4) concomitantly with PPI	24
Brancato et al. (33)	2014	Prospective	Hypothyroid patients with elevated TSH on tablet LT4	53
Fallahi et al. (34)	2016	Prospective	Patients with subclinical hypothyroidism treated with tablet LT4 not reaching optimal TSH despite not known causes of malabsorption	21
Benvenga et al. (35)	2017	Prospective	Patients with tablet LT4 malabsorption caused by calcium and/or iron supplements	19
Fallahi et al. (36)^a^	2017	Prospective	Hypothyroid patients, treated with tablet LT4, undergone bariatric surgery of Roux-en-Y gastric bypasses	13
Vita et al. (37)	2017	Prospective	Patients with T4 malabsorption due to concomitant use of multiple drugs	11

*Articles appear in the table by the year of publication. All studies switched patients from tablet to liquid LT4 at unchanged dose*.

*^a^This study described two subgroups of patients, and we excluded that of four cases according to the studies selection criteria (see Materials and Methods)*.

### Risk of Bias

Risk of bias was assessed by four issues and results of each study reported in Figure 2. Three papers had a study design showing higher risk of bias, other three were at risk for their reporting, and one paper was assessed at higher risk of carryover.

## Discussion

### Summary of Findings

Here, we systematically reviewed the available literature on the use of liquid solution LT4 in patients previously treated with tablet LT4. Our search allowed retrieving six studies reporting data about 141 patients. Notably, all these articles described a significant decrease of TSH levels after switching from tablet to liquid LT4, leaving the dose unchanged. Analyzing the pooled results, we found that the mean difference of TSH decrease was 4.23 mIU/L, and this finding showed a strong statistical significance. The six studies included in our meta-analysis enrolled patients who switched to liquid LT4 because of different reasons, mainly represented by malabsorption; in the manuscripts included, the authors selected their patients with suboptimal TSH, switched LT4 preparation, and obtained the improvement of serum TSH values. All these data suggest that the use of liquid LT4 preparation can reduce TSH levels obtained with the same dose of tablet LT4 and the present meta-analysis reinforces this evidence.

Some other papers initially eligible but afterward excluded from our study (21, 26–31) gave important information about this topic: almost all articles described the efficacy on reaching optimal TSH values in patients previously undertreated with tablet T4 formulation as depicted in Table 2. Interestingly, the paper describing the largest series of patients (27) showed the reduction of TSH levels after the switch to liquid solution even in patients without malabsorption and already with optimal serum thyroid balance on tablet LT4. Noteworthy, in a study (21) enrolling only highly selected and cured patients with differentiated thyroid cancers, switched from tablet to liquid LT4, it has been shown a significant reduction of subjective symptoms with overlap of therapeutic effectiveness. This finding might indirectly prompt physicians to use a dose of liquid LT4 lower than one could estimate with tablet formulation, similarly to that proposed for softgel preparation in a recent study (38).

**Table 2:** Overview of the eligible studies subsequently excluded from the present review.

Reference	Year	Study type	Patients type	Patients who completed the study (*n*)
Pirola et al. (26)	2014	Prospective	Hypothyroid patients, treated with tablet levothyroxine (LT4), undergone bariatric surgery	4
Giusti et al. (21)	2014	Prospective	Thyroidectomized for differentiated thyroid cancer	54
Fallahi et al. (27)	2016	Prospective	Patients with optimal TSH values during tablet T4 treatment	141
Fallahi et al. (28)	2016	Prospective	Patients with autoimmune atrophic gastritis	5
Fallahi et al. (29)	2017	Prospective	Patients with lactose intolerance	5
Peirce et al. (30)	2017	Prospective	Thyroidectomized patients	3
Hommel and Delgrange (31)	2017	Prospective	Hypothyroid patient, treated with tablet LT4, undergone bariatric surgery	1

*Articles appear in the table by the year of publication. All studies switched patients from tablet to liquid LT4 at unchanged dose. These studies were excluded from our analysis according to the studies selection criteria (see Materials and Methods)*.

### Limitations

The reliability of liquid LT4 in reducing TSH levels of tablet T4-treated patients should be ideally evaluated with randomized controlled trials (RCT) with a cross-over design. One RCT was published on the use of liquid LT4 and proved the efficacy of this formulation, even in the case of T4 ingestion concomitant with breakfast (25), independently from its composition. The same result has been described in a crossover study by Morelli et al. (22). The six papers found by the present systematic review had a design quite similar to a cross-over study. A selection bias might be present in this meta-analysis because the included papers did not select patients prior to begin tablet LT4 treatment. In fact, the patients enrolled were already on tablet LT4 treatment, because these studies were aimed at comparing the performance of liquid over tablet LT4 in improving suboptimal TSH of these patients. Also, this design shows several advantages: each participant acted as her/his own control eliminating among-participant variation, addressing the problem of statistical power, and every participant received both interventions. The main problem associated with this study design is that of carryover, such as the situation in which the effects of the first intervention given (tablet LT4 in our case) keep on the subsequent period, thus interfering with the effects of the second intervention (liquid LT4). To skip this problem, some cross-over trials may decide to include a period between interventions known as a washout period. However, here, we are confident to exclude a carryover effect because we considered in our analysis only TSH value on tablet LT4 and 2 months after the switch to liquid formulation. Among other potential limitations of the present data, we meta-analyzed here studies published very recently and with no large sample size. Generally, studies reporting preliminary results, in small series, and with positive findings are more likely to be published. As a final remark, only Italian authors published on this topic due to the large availability of liquid LT4 in our country. These findings have to be verified in other countries and in populations with different habits, which could influence the effect of different thyroxine preparations.

### Conclusion

In conclusion, the present meta-analysis supports the evidence that, in tablet LT4-treated patients with suboptimal TSH, one can obtain a lower TSH value by switching therapy to liquid LT4 formulation at unchanged dose. This may be of interest in clinical practice in patients with refractory hypothyroidism.

## Ethics Statement

This article does not contain any studies with human participants or animals performed by any of the authors.

## Author Contributions

CV, PT, and LG conceived and designed the study. CV, MC, AA, PF, and MS performed the literature search; PT analyzed the data; MC, AA, LG, PF, and MS critically reviewed the results. All seven authors contributed to write the manuscript.

## Conflict of Interest Statement

The authors declare that the research was conducted in the absence of any commercial or financial relationships that could be construed as a potential conflict of interest. The review editor SF declared a shared affiliation, though no other collaboration, with the authors PF and AA.
